# Identification of Synaptosomal Proteins Binding to Monomeric and Oligomeric α-Synuclein

**DOI:** 10.1371/journal.pone.0116473

**Published:** 2015-02-06

**Authors:** Cristine Betzer, A. James Movius, Min Shi, Wei-Ping Gai, Jing Zhang, Poul Henning Jensen

**Affiliations:** 1 University of Aarhus, DANDRITE—Danish Research Institute of Translational Neuroscience & Department of Biomedicine, Aarhus, Denmark; 2 Flinders University School of Medicine, Department of Human Physiology and Centre for Neuroscience, Bedford Park, SA, Australia; 3 Washington School of Medicine, Department of Pathology, Seattle, United States of America; University of Padova, ITALY

## Abstract

Monomeric α-synuclein (αSN) species are abundant in nerve terminals where they are hypothesized to play a physiological role related to synaptic vesicle turn-over. In Parkinson’s disease (PD) and dementia with Lewy body (DLB), αSN accumulates as aggregated soluble oligomers in terminals, axons and the somatodendritic compartment and insoluble filaments in Lewy inclusions and Lewy neurites. The autosomal dominant heritability associated to mutations in the αSN gene suggest a gain of function associated to aggregated αSN. We have conducted a proteomic screen to identify the αSN interactome in brain synaptosomes. Porcine brain synaptosomes were fractionated, solubilized in non-denaturing detergent and subjected to co-immunoprecipitation using purified recombinant human αSN monomers or oligomers as bait. The isolated αSN binding proteins were identified with LC-LTQ-orbitrap tandem mass spectrometry and quantified by peak area using Windows client application, Skyline Targeted Proteomic Environment. Data are available via ProteomeXchange with identifier PXD001462. To quantify the preferential binding an average fold increase was calculated by comparing binding to monomer and oligomer. We identified 10 proteins preferentially binding monomer, and 76 binding preferentially to oligomer and a group of 92 proteins not displaying any preferred conformation of αSN. The proteomic data were validated by immunoprecipitation in both human and porcine brain extracts using antibodies against monomer αSN interactors: Abl interactor 1, and myelin proteolipid protein, and oligomer interactors: glutamate decarboxylase 2, synapsin 1, glial fibrillary acidic protein, and VAMP-2. We demonstrate the existence of αSN conformation selective ligands and present lists of proteins, whose identity and functions will be useful for modeling normal and pathological αSN dependent processes.

## Introduction

The neurodegenerative α-synucleinopathies are dominated by PD, DLB, and multiple system atrophy (MSA), which are characterized by aggregation and deposition of αSN [1, 2]. αSN is a 14 kDa natively unfolded protein, and *in vitro* studies have shown that monomeric, soluble oligomeric, and insoluble fibrillar species of αSN exists in equilibrium [3–5]. αSN is normally located in the presynaptic nerveterminals in high concentrations [6]. The function of αSN in the nerve terminal remains ambiguous, but recent reports suggest a functional role as chaperone for the SNARE complex [7]. αSN is causally linked to autosomal dominant forms of PD where missense mutations in αSN (A30P, E46K, A53T, H50Q, and G51D) [8–12] and multiplications of the normal αSN coding reading frame cause autosomal dominant PD and DLB [13, 14]. Moreover, GWAS studies have identified variation in the αSN gene as the strongest genetic risk factor for sporadic PD [15–17], and substitutions of A18T and A29S have been associated with sporadic PD [18].

The autosomal dominant disease associated mutations of αSN suggest a gain of toxic function and the soluble αSN oligomers are hypothesized to represent the culprit (Reviewed in [19, 20]) based on biophysical, cellular and *in vivo* studies in models and man [21–24]. Evidence suggests αSN aggregation begins in nerve terminals and spread from synapses via axons to cell bodies forming Lewy neurites and Lewy bodies [25]. Hence, presynaptic proteins that interact with aggreated αSN species may represent the first neuronal partners in protein networks that are off-set by early αSN aggregates and recent data on prion-like spreading of αSN pathology in mouse suggest a role for transsynaptic spreading of αSN pathology [26, 27].

Mass spectrometry based proteomic analyses are powerful tools to profile changes in proteins and identify protein-protein interactions in biological systems [28, 29]. αSN has previously been the target for studies of its presence and post translational modifications [30–32]. αSN binding partners have been investigated in co-immunoprecipitation experiments in cell extracts, e.g. subjected to oxidative stress [33, 34] and in brain extracts for Ser-129 phosphorylation dependent interactions and targets for insoluble αSN filaments [35, 36].

To investigate the interactome disease-related oligomeric αSN in a preparation as close to its normal subcellular localization we used purified porcine brain synaptosomes. We conducted semi-quantitative comparison of the interactions for monomeric and oligomeric αSN to get an impression on the potential gain-of-function caused by the process of aggregation. The αSN oligomers used in this screen share structural similarities with oligomer species present in human dementia with Lewy body brain tissue and species developing in αSN transgenic mouse at the time of degeneration [21, 22]. Synaptosomes have classically been used as the starting material for purifying synaptic vesicles because they are enriched in axonal nerve terminals, but also contains mitochondria, myelin and other brain cell structures [37]. Because sample complexity and the wide dynamic range of concentrations of analytes counteracts the ability of our MS based strategy to characterize the potential αSN interactome proteome, we reduced sample complexity by subfractionating the crude synaptosomal preparation into synaptosomal membranes, synaptosomal lysate, synaptic vesicles, and cytosol prior to non-denaturing detergent extraction [38]. The extract was incubated with purified αSN monomers or oligomers prior to co-immunoprecipitation (Co-IP) using anti-αSN antibodies. The interacting proteins were identified by label-free proteomic analysis on a LC-LTQ-orbitrap tandem mass spectrometer followed by a semi-quantitative analysis using Skyline Targeted Proteomic Environment [39]. Based on the quantitative analysis, we could group the ligands in three classes of interacting proteins, preferential monomeric (MP), preferential oligomeric (OP), and αSN interacting proteins with no detectable conformational preferences (NPB). Among the ligands were proteins involved in pathways genetically associated to PD and functionally linked to αSN dependent degeneration, e.g. VAMP-2. The identification of αSN targets and their semiquantitative binding preference for monomeric versus oligomeric αSN bring information that can substantiate current and and help build future hypotheses and models.

## Materials and Methods

### Production and isolation of αSN monomer and oligomer

**Figure 1:**
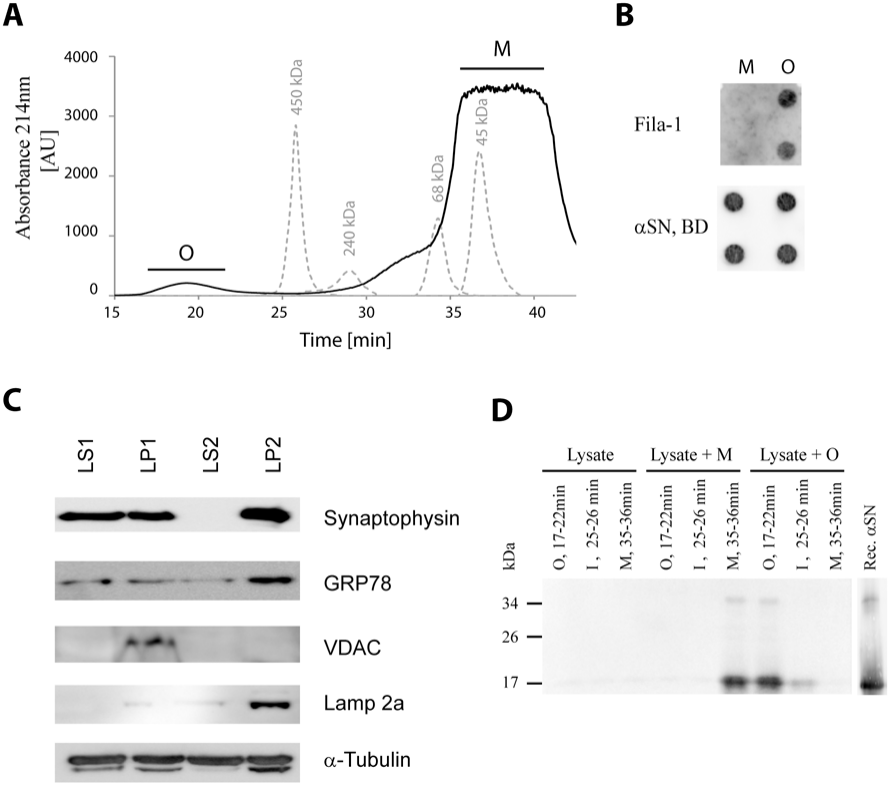
Isolation and characterization of αSN monomers, oligomers, and synaptosomal fractions for co-immunoprecipitation experiments. (A) αSN oligomers formed by reconstitution of lyophilized monomeric recombinant human αSN was isolated by gelfiltration. Oligomers (O) were collected from 17–22 min, and monomer αSN was collected at 35 min. The dashed line shows chromatogram of globular protein standards, ferritin (450 kDa), catalase (240 kDa), albumin (68 kDa), and ovalbumin (45 kDa). (B) The misfolded state of the oligomers was demonstrated by dot blotting of 250 ng αSN monomer and oligomer in duplicates using the aggregate-specific antibody Fila-1 and the total loading of αSN on the filter by pan α-synuclein antibody (αSN BD). (C) Characterization of the porcine synaptosomal fractions used for pull-down experiments. 30µg from synaptosomal membranes (LP1), synaptosomal lysate (LS1), synaptic vesicles (LP2) and synaptosomal cytosol (LS2) were immunoblottet and presence of specific markers for synaptic vesicle synaptophysin, endoplasmic reticulum 78 kDa glucose-regulated protein (GRP78), mitochondria voltage-dependent anionic channel (VDAC), lysosomal LAMP-2a, and α-tubulin. (D) The stability of the isolated αSN monomer and oligomer in brain fractions during the co-immunoprecipitation protocol was studied by a subsequent gel filtration. Detergent extracts of porcine brain (LS2) was incubated with buffer as negative control (Lysate), monomer (Lysate + M) and oligomer (Lysate + O) for 24 h at 4oC, followed by gelfiltration. Samples from fractions eluting corresponding to O, M and a fraction eluting at 25 min between monomer and oligomer were resolved by SDS-PAGE and subjected to immunoblotting with monoclonal anti-α-synuclein antibody (αSN BD). The nature of αSN does not change either as demonstrated by the blot of recombinant monomeric αSN. Clearly the monomer migrates in the M fraction and the oligomer predominantly in the O fraction. The level of endogenous porcine αSN is low compared to exogenous recombinant αSN.

Human recombinant αSN was produced as described previously [40]. In order to prepare monomer and oligomer, lyophilized αSN was dissolved in PBS (7.2 mM Na_2_HPO_4_, 2.8 mM NaH_2_PO_4_, 140 mM NaCl, pH 7.4) to a final concentration of 10 mg/ml (690µM) and incubated for 30 min on ice. The dissolved protein was centrifuged on a tabletop centrifuge at 14,800 rpm for 5 min at 4°C and afterwards loaded on a Superdex 200 10/30 GL column (GE Healthcare) and eluted with 0.5 ml/min PBS [41]. Oligomers were collected from 17–22 min, and monomer αSN was collected at 35 min (Fig. 1) Protein concentrations of the fractions were determined by Bicinchoninic acid protein concentration assay (BCA) (Pierce).

### Preparation of synaptosomal lysates from porcine brain

The synaptosomal fractions were prepared as described previously [42], with minor modifications. A schematic flowchart of the preparation of the synaptosomal fractions can be found in S1 Fig. After collecting fresh porcine brain tissues (Jensen’s Slagtehus, DK), all steps were carried out on ice or at 4°C. The cortical grey matter region was dissected to avoid myelin-rich areas. The harvested tissue was homogenized in 3 x volume (w/v) ice-cold homogenization buffer (320 mM sucrose, 4 mM HEPES–NaOH, 2 mM EDTA, and Complete protease inhibitor mix (Roche), pH 7.4) using a loose-fitting glass-Teflon homogenizer (10 up-and-down strokes, 700 rpm). The homogenate was centrifuged for 10 min at 1000 *G* in a Sorvall RC 5C plus centrifuge. The resulting pellet (P1) was discarded, while the supernatant (S1) was collected and centrifuged for 15 min at 12 000 *G*. The supernatant (S2) was removed, and the pellet (P2) was washed by resuspension in 30 mL of homogenization buffer and recentrifuged for 15 min at 13 000 *G* to yield a supernatant, S2′, and a pellet, P2′. The latter pellet represents a crude synaptosomal fraction, which was subsequently resuspended in homogenization buffer, then homogenized in a glass-Teflon homogenizer. 10 x (v/v) ice-cold water containing protease inhibitors were added, and the whole suspension was immediately subjected to five up-and-down strokes at 1000 rpm. (hypotonic lysis). 1 M HEPES–NaOH buffer (pH 7.4) was immediately added to a final concentration of 10 mM HEPES followed by 30 min incubation on ice. The lysate was centrifuged for 20 min at 33 000 *G* to yield the lysate pellet (LP1—synaptosomal membranes) and a lysate supernatant (LS1—synaptosomal lysate). Subsequently, the supernatant was centrifuged for 2 hours at 260 000 *G* in Beckman Optima LE-80K ultracentrifuge. The supernatant was isolated (LS2—high speed cytosol) and pellet (LP2—synaptic vesicles) was resuspended in homogenization buffer. Complete protease inhibitor (Roche) and Triton X-100 to a final concentration of 0.5% were added to all samples.

### Preparation of synaptosomal lysates from human brain

The human brain tissue from an 80 year old neurological intact male patient was from the South Australian Brain bank. The synaptosomal fractions were prepared as described for the porcine brain.

### Co-immunoprecipitation of αSN binding proteins

2 µg purified αSN monomer or αSN oligomer was incubated with 1 mg/ml synaptosomal lysate, synaptosomal membranes, synaptic cytosol, or synaptic vesicles in PBS, 0.5% Triton X-100 in a total volume of 1 ml at 4°C in rotating tubes overnight. Affinity purified rabbit poly-clonal αSN specific antibody (ASY-1) covalently bound to Sepharose beads 4B (GE Healthcare) was added in a 5 times molar excess (5 IgG: 1 αSN) to the samples followed by 2 hours incubation at 4°C in rotating tubes. The Sepharose beads were isolated and washed twice with PBS, 0.5% Triton X-100, and Co-IP proteins were eluted by incubation in non-reducing SDS loading buffer for 2 hours at room temperature. Three individual Co-IP were conducted in the four described synaptosomal preparations using either monomeric, oligomeric αSN, or buffer as negative control, resulting in 36 samples to be analyzed by mass spectrometry.

### Mass spectrometric peptide mapping

Samples were tryptic digested, and analyzed by LC-LTQ-Orbitrap mass spectrometry as previously described [43] but with minor modifications. Briefly, digested samples were acidified with 0.1 volume of 10% formic acid and cleaned using Ultra Microspin columns (Cat# SUM SS18V, The Nest Group, Southborough, MA, USA) following manufacturer’s instructions. Samples were then dried to completion in a rotor speed vacuum and resuspended in 80 µl of 98% ddH_2_O, 2% acetonitrile, 0.1% formic acid for analysis. Liquid chromatography was performed using a Waters NanoAcquity UPLC (Waters Corporation, Milford, MA). Peptides were separated online with 75 µm i.d. × 20 cm home-packed fused silica columns (100 Å Magic C18AQ: Michrom Bioresources, Auburn, CA, USA) with a 125 min 5–90% acetonitrile/water gradient containing 0.1% formic acid. All data were acquired on a LTQ-Orbitrap XL mass spectrometer (Thermo Corporation, San Jose, CA). Data Dependent Acquisition mode was employed for this instrument using a “Top 10” method and 125-minute acquisition time. The high resolution (mass accuracy: 5 ppm or less) MS data was acquired (60,000 at 400 *m/z*) followed by 10 ion trap MS/MS acquisitions. The AGC target value for the precursor scan was 1E6 counts and for the ion trap MS/MS scans to 3E4 counts at a minimum MS signal level of 2000 counts and an isolation window of 1.6 *m/z*. Charge state screening was considered for all MS/MS targets, utilizing only +2,+3, and +4 charge state isotope distribution. Dynamic exclusion was used with parameters set at 1 repeat count, 30 second repeat duration, 500 item exclusion list size, and an exclusion duration of 180 seconds. The mass spectrometry proteomics data have been deposited at the PRIDE partner repository (dataset identifier PXD001462) of the ProteomeXchange Consortium (http://proteomecentral.proteomexchange.org) [44].

### Semi-quantitative identification of proteins based on mass spectrometric peptide mapping

RAW data files were converted to mgf and mzXML files using MS Convert (Proteowizard, v3.0.3951). The mgf were then searched using ProteinPilot v4.1 (AB SCIEX, Framingham, MA, USA) referencing a *Sus Scrofa* database containing all canonical sequences plus all manually reviewed isoform sequences retrieved from UniProt (July 2012; 26,685 protein entries) for peptide and protein identification. Default settings for peptide assignments and protein identifications were used (e.g., Identification as the Sample Type, Biological modifications as the ID Focus, and Unused ProtScore of 0.05 as the Detected Protein Threshold). In case that multiple proteins are identified by the software as equivalent “winners” (i.e., they share all or nearly all of the same peptides identified in the sample and are usually different isoforms in a protein family), the first one is arbitrarily chosen as the representative winner presented in the tables. The mzXML files were searched using X Tandem! Database searching as part of the TransProteomics Pipeline (Institute for Systems Biology) to create spectral libraries; static carbamidomethyl modifications to cysteine and variable oxidation of methionine, as well as allowance for two missed tryptic cleavages were considered. The resulting ~pep.xml result files were grouped according to experimental condition in Skyline (v 1.3, MacCoss Lab, Department of Genome Sciences, University of Washington, Seattle, WA, USA) to create distinct spectral libraries. 36 spectral libraries were generated from the three biological replicates of Co-IP from the four different synaptosomal preparations (LP1, LS1, LP2, LS2) using the three different baits (PBS, monomer, and oligomer). All peptides from these libraries were added to the project to populate the peptide list, resulting in 11087 peptides derived from 2049 *Sus scrofa* proteins. The 36 RAW files from the experimental isolation conditions were then imported to create the MS1 Extracted Ion Chromatograms (XIC) for each peptide. From the spectral libraries, the identification retention time was indicated on these XICs where identification was made from the database search for that RAW file. This was done in Skyline by making connections between the RAW filename and the pep.xml filename in the spectral library. All peptide XICs were manually inspected and only peptides that had at least two positive identifications were retained. Additionally, peak assignments were manually corrected for all files based on these identification retention times, ensuring the correct MS1 signal was integrated. To further validate the specificity of these peak assignments, XICs were created in Skyline for all precursor isotopes, a predicted isotopic distribution of the peptide sequence was then calculated, and this predicted ratio was compared with the actual isotopic ratios for the MS1 signal of all peaks. From this, an ‘idotp’ dot product number, where 1.00 is a perfect match, was generated in Skyline. These idotp values were also considered for peak assignment. Identified proteins were pre-filtered by excluding proteins with higher peak areas in buffer controls than both monomer and oligomer samples. Peak areas of the remaining proteins were logarithmic transformed in order to reduce the variability between the biological replicates and normalized to averaged peak areas of buffer-control. The average fold increase was calculated as the ratio between peak areas for monomer and oligomers for monomer preferential binding proteins and as ratio between peak areas for oligomers and monomers for preferential oligomer binding proteins. In order for a protein to be considered as either monomer or oligomer binding Student’s t-test was applied and conventional significance of 95% was accepted. The criteria of ≥ 2-fold increase in average fold increase has to be fulfilled in order to be considered as preferential binding protein, similar to the criteria set up in the phospho-peptide interaction study by McFarland *et al*. [35]. Based on the filtering αSN preferential monomer binding proteins are listed in Table 1, and preferential αSN binding proteins are listed in Table 2. Proteins with significant higher binding to αSN compared to buffer are listed as non-conformation specific αSN binding proteins in Table 3. The porcine proteome is not completely described and unknown proteins are listed with the predicted name derived from an Ensembl automatic analysis pipeline. A flowchart of how quantitative data was obtained from sample preparation to semi-quantitative analysis including criteria for exclusions/inclusion is presented in S2 Fig., and semi-quantitative data can be found in S1 Table along with number of identified peptides, number of quantified peptides, and percent sequence coverage.

**Table 1:** Preferential αSN monomer interacting proteins.

**Protein**	**UniProt/ swiss-Prot**	**Synapto-somal** **fraction**	**Fold** **increase**	**p-value**
***Mitochondria***				
Cytochrome b-c1 complex subunit 2	F1RPD2_PIG	LP2	16	<0.03
Stomatin protein 2	F1SIH5_PIG	LP1	5	0.02
GABA aminotransferase(4-aminobutyrate aminotransferase)	GABT_PIG	LP1	23	<0.01
Fumarylacetoacetate hydrolase domain-containing protein 2	F1SU52_PIG	LP1	6	<0.01
***Plasma membrane***				
Myelin proteolipid protein*	MYPR_PIG	LS1	3	0.03
***Cytoplasm***				
Abl interactor 1*	F1RTW7_PIG	LP2	2	<0.01
Phosphatidylethanolamine-binding protein 1	F1RKG8_PIG	LP1	16	<0.01
TNF receptor-associated protein 1	F1RK45_PIG	LP1	6	0.02
Tropomodulin-2	F1RZB5_PIG	LP2	8	0.01
***Vesicle***				
V-type proton ATPase subunit F	F1SMN6_PIG	LP1	9	<0.01

Co-immunoprecipitations of proteins from detergent extracts of porcine synaptosomal fractions using recombinant αSN monomer, αSN oligomer, or buffer as bait, were conducted, and the interacting proteins were identified by LC-LTQ orbitrap tandem mass spectrometry. The identified proteins are specified by name, Uniprot/swiss-Prot entry, and the synaptosomal fraction in which they were identified. Quantitative analyses were based on Peak Area Intensity of peptides occurring in at least two of three individual immunoprecipitations. The average fold increase is calculated as the ratio between the peak areas for monomer and oligomers for monomer preferential binding proteins. The criteria of ≥ 2 fold increase compared to oligomer had to be fulfilled in order to be considered a preferential αSN monomer binding protein. In order for a protein to be considered as either monomer or oligomer binding Student’s t-test was applied to the logarithmic transformed peak areas and significance < 0.05 was accepted.

* indicates the monomer preferential proteins that were validated by western blotting in Fig. 3.

**Table 2:** αSN oligomer interacting proteins.

**Protein**	**UniProt/ swiss-Prot**	**Synapto-somal** **fraction**	**Fold increase**	**p-value**	**Protein**	**UniProt/ swiss-Prot**	**Synapto-somal** **fraction**	**Fold increase**	**p-value**
***Mitochondrial***									
3-hydroxyacyl-CoA dehydrogenase type-2	F1RUI1_PIG	LP1	6	0.01	2-oxoglutarate/malate carrier protein	F1RFX9_PIG	LP2	4	0.05
ADP/ATP translocase 3	ADT3_PIG	LS1	66	<0.01	Glutamate carrier 1	F1RYY8_PIG	LS1	49	<0.01
ATP synthase subunit α	F1RPS8_PIG	LS1	13	<0.01	Mitochondrial import inner membrane translocase subunin	F1SA66_PIG	LS2	3	0.01
Ca^2+^-binding mitochondrial carrier protein Aralar2	I3L614_PIG	LS1	17	0.01	Phosphatidate cytidylyl-transferase 2	I3LAQ3_PIG	LS1	5	<0.01
Elongation factor Tu	F1RFI1_PIG	LP1	22	<0.01	Pyruvate carboxylase	F1RUV6_PIG	LS1	7	0.01
Heat shock 60 kDa protein 1	F1SMZ7_PIG	LS2	22	<0.01	Heat shock 70kDa protein 9 (Mortalin)	F1RGJ3_PIG	LS1	2	<0.02
LETM1 and EF-hand domain-containing protein 1	F1S6V4_PIG	LS2	60	<0.01	Trifunctional enzyme α	ECHA_PIG	LS1	7	<0.01
***Endoplasmic reticulum***									
78 kDa glucose-regulated protein	F1RS36_PIG	LP2, LS2	3	0.03	Hypoxia up regulated protein 1	F1SAI8_PIG	LP2	4	0.04
Endoplasmin	ENPL_PIG	LS1	4	0.01	Reticulocalbin 2	F1SJ93_PIG	All	15	<0.01
***Plasma membrane***									
Excitatory amino acidtransporter 1	F1SNA0_PIG	LS1, LP2	4	0.03	Plasma membrane Ca^2+^ ATPase 4	F1S6B3_PIG	LS1	6	<0.01
Excitatory amino acidtransporter 2	F1SHF9_PIG	LS1	7	<0.01	Syntaxin-binding protein 1	F1RS11_PIG	LS1, LS2	23	0.05
				Transmembrane protein 33	F1S4G6_PIG	LS1	399	<0.01
***Cytoplasm***									
40S ribosomal protein S3	RS3_PIG	LS2	7	<0.01	Dynein 1, light intermediate chain 2	I3LQU0_PIG	LS2	22	<0.01
40S ribosomal protein S5-like isoform 2	F2Z5E6_PIG	LP2	5	0.02	Glial fibrillary acidic protein*	F1RR02_PIG	LS1	3	<0.01
60S ribosomal protein L32	RL32_PIG	LS1	19	<0.01	Glutamate decarboxylase 2*	DCE2_PIG	LS2	38	<0.01
52 kDa Rho protein	F1RHN8_PIG	LS2	2	0.03	Neuronal migration protein doublecortin	I3L5C8_PIG	LS1, LS2	53	<0.01
α-crystallin B chain	CRYAB_PIG	LP2	3	0.01	Rab GDP dissociation inhibitor α	I3L893_PIG	LS1	8	0.01
Aminoacyl tRNA synthase complex-interacting multifunctional protein 2	F1RFM7_PIG	LP2	10	0.01	Ribosomal protein, large, P2	F1RYZ0_PIG	LS1	11	<0.01
ATP-citrate synthase	F1S0N1_PIG	LS2	58	0.02	S100 Ca^2+^ binding protein A14	F1SFV3_PIG	LS1	3	0.01
Ca^2+^-binding protein 1	F1RJI3_PIG	LP2	10	0.01	Seryl-tRNA synthetase	F1S5Z3_PIG	LS2	261	<0.01
cAMP-dependent protein kinase catalytic β	KAPCB_PIG	LP2	5	0.01	T-complex protein 1 α	F1SB63_PIG	LS2	373	<0.01
Chaperonin containing TCP1, subunit 2β	D0G0C8_PIG	LS2	478	<0.01	T-complex protein 1 δ	F1SQN1_PIG	LS1	11	0.01
Dynein 1 intermediate chain 2	F1S087_PIG	LS1	10	<0.01	T-complex protein 1 η	F1SLF6_PIG	LS1	133	0.03
FMR1-interacting protein 2	F1RQE9_PIG	LP2	3	0.02	T-complex protein 1 γ	F1RP17_PIG	LS1, LS2	204	<0.01
Dihydropyrimidinase 2	I3LJE2_PIG	LS1, LS2	23	<0.01	14–3–3 protein ζ/δ	F2Z558_PIG	LP1	2	0.04
Dynein heavy polypeptide	F1S9Y5_PIG	LS2, LP2	802	<0.01	14–3–3 protein γ	F2Z4Z1_PIG	LP1	9	0.03
***Nucleus***									
A-kinase anchor protein 5	F1SA75_PIG	LS1	9	0.01	Signal recognition particle 54 kDa protein	F2Z5M9_PIG	LS2	18	<0.01
***Vesicle***									
Amphiphysin	I3L8X6_PIG	LP2	5	0.03	Synaptic vesicle glycoprotein 2A	F1SDF9_PIG	LS1	9	<0.01
Nipsnap homolog 1	F1RFF5_PIG	LP1	3	0.03	Tripeptidyl-peptidase 1	I3L812_PIG	LS2	45	<0.01
Sorting nexin 6	F1SHH3_PIG	LS2	181	<0.01	Vacuolar protein sorting-associated protein 53	F1RHI3_PIG	LP2	31	<0.01
Synapsin 1*	B7TY10_PIG	LS1, LS2, LP2	9	0.01	V-type proton ATPase subunit d 1	F2Z5H6_PIG	LP2	2	0.03
***Cytoskeleton***									
α-centractin	F2Z5G5_PIG	LS2	20	0.02	Spectrin α chain	F1RR78_PIG	LS1	3	<0.01
α-internexin	F1S847_PIG	LS1	3	0.02	Tubulin α-4A chain	F2Z5S8_PIG	LS1	6	0.01
Dynamin-2	F1S593_PIG	LS2	64	<0.01	Tubulin β-3 chain	F1S6M7_PIG	LS1, LS2	15	0.01
Microtubule-associated protein 6	F1SUM1_PIG	LS1, LS2	28	<0.01	Tubulin β-4 chain	F2Z5K5_PIG	LS1, LS2, LP2	9	0.01
Neurofilament heavy polypeptide	F1RFH3_PIG	LP1	4	0.02	Tubulin polymerization-promoting protein	I3LB30_PIG	LP1	3	0.04
Sirtuin 2	I3L8A1_PIG	LS1	66	0.01					
***Secreted***									
Complement C4 precursor	F1RQW2_PIG	LS1	2	0.01	C-type natriuretic peptide	F1S0P3_PIG	LS1, LP2	7	0.05
Complement C5 precursor	F1SME1_PIG	LS2	19	<0.01	Tenascin-R	F1S706_PIG	LS2	8	0.01

Co-immunoprecipitations of proteins from detergent extracts of the synaptosomal fractions from porcine brain using recombinant αSN monomer, αSN oligomer, or buffer as bait, were conducted, and the interacting proteins were identified by LC-LTQ orbitrap MS/MS. The identified proteins are specified by name, Uniprot/swiss-Prot entry, and the synaptosomal fraction in which they were identified. Quantitative analyses were based on Peak Area Intensity of peptides occurring in at least two of three individual immunoprecipitations. The average fold increase is calculated as the ratio between the peak areas for monomer and oligomers for oligomer preferential binding proteins. The criteria of ≥ 2 fold increase compared to monomer had to be fulfilled in order to be considered a preferential αSN oligomer binding protein. In order for a protein to be considered as either monomer or oligomer binding Student’s t-test was applied to the logarithmic transformed peak areas and significance < 0.05 was accepted.

* indicates the oligomer preferential proteins that were validated by western blotting in Fig. 3.

**Table 3:** Non-conformation specific αSN interacting proteins.

**Protein**	**UniProt/ swiss-Prot**	**Synaptosomal**	**Protein**	**UniProt/ swiss-Prot**	**Synaptosomal**
***Mitochondrial***					
2-oxoglutarate dehydrogenase	F1SSH8_PIG	LP2	Mitochondrial carrier homolog 2	F1SIE0_PIG	LP1
39S ribosomal protein L12	I3LSY1_PIG	LP2	Mitochondrial import inner membrane translocase subunit	F1RK50_PIG	LP1
Adenine nucleotide translocator 2	F2Z565_PIG	LS1, LP1	NADH dehydrogenase [ubiquinone] 1 β subcomplex subunit 10-like	I3LDC3_PIG	LP1
ATP synthase subunit beta	F1SLA0_PIG	LP2	NADH dehydrogenase [ubiquinone] flavoprotein 1	F1RVN1_PIG	LP2
Cytochrome b-c1 complex subunit 8-like isoform 2	F1RI18_PIG	LP2	NADP-dependent malic enzyme	F1STS4_PIG	LP1
Cytochrome c oxidase subunit 1	COX1_PIG	LP2	Pentatricopeptide repeat domain 3	F1SVC4_PIG	LP2
Chymodenin	F1RLH7_PIG	LP2	Prohibitin 2	I3LQN4_PIG	LP1, LP2
Dihydrolipoyllysine-residue acetyltransferase component of pyruvate	F1SMB2_PIG	LP1, LP2	Pyruvate dehydrogenase: subunit beta	F1SGH5_PIG	LP2
Dihydrolipoyllysine-residue succinyltransferase component of 2-oxoglutarate dehydrogenase complex	ODO2_PIG	LP1, LP2	Short-chain specific acyl-CoA dehydrogenase	F1RJH2_PIG	LP1
Glutamate dehydrogenase 1	DHE3_PIG	LP1	Thioredoxin-dependent peroxide reductase	F1S418_PIG	LS1
Hexokinase 1	F1SUF2_PIG	LP2	Translocase of outer mitochondrial membrane	F2Z4X6_PIG	LP1
Long-chain 3-ketoacyl-CoA thiolase	F1SDN2_PIG	LP1	Voltage-dependent anion-selective channel 1	VDAC1_PIG	LP1, LP2
***Endoplasmic reticulum***					
B-cell receptor-associated protein 31	F1S2A8_PIG	LP2	Phospholipase D3	I3L5D6_PIG	LP2
Ca^2+^/calmodulin-dependent protein kinase II γ-B	Q7JFN4_PIG	LP2	Protein disulfide-isomerase A6	E1CAJ6_PIG	LP2
NADH dehydrogenase (ubiquinone) Fe-S protein 5, 15 kDa (NADH-coenzyme Q reductase)	F1SV23_PIG	LP1, LP2	Stromal interaction molecule 1 precursor	F1SUZ4_PIG	LP2
***Plasma membrane***					
Adaptor-related protein complex 2, mu 1 subunit	I3LL07_PIG	LP2	Immunoglobulin superfamily member 8	F1RJW5_PIG	LP2
AP-2 complex subunit β	I3L6Y6_PIG	LP2	Neural cell adhesion molecule 1	F1SM72_PIG	LP1, LP2
BPI fold-containing family B member 1	I3LAK0_PIG	LP2	Neurotrimin	F1S6D0_PIG	LP2
Brain acid soluble protein 1 isoform 1	F1SRL9_PIG	LP1	Oligodendrocyte-myelin glycoprotein	F1RJ55_PIG	LP2
Ca^2+^/calmodulin-dependent protein kinase II α	F1RL74_PIG	LP2	Paralemmin-1	PALM_PIG	LP2
Ca^2+^ modulating ligand	F1RHC6_PIG	LP2	Plasma membrane Ca^2+^ ATPase 2	I3LIE6_PIG	LP2
Cell adhesion molecule 2	F1SK66_PIG	LP2	Prohibitin	F2Z543_PIG	LP2
Cell cycle exit and neuronal differentiation protein 1	CEND_PIG	LP1	Protein RER1	I3LJC8_PIG	LP2
Cytolytic trigger molecule G7	FCGR3_PIG	LP1	Protein transport protein Sec61 subunit α2	F2Z5D0_PIG	LP2
EH domain-containing protein 3	F1RQR4_PIG	LP2	Thy-1 membrane glycoprotein precursor	B9ZSM8_PIG	LP2
***Cytoplasm***					
40S ribosomal protein S3a	F2Z5C7_PIG	LP2	Elongation factor 1-α	Q0PY11_PIG	LP2
60S ribosomal protein L6	RL6_PIG	LP2	Elongation factor 1-β	F1SHD6_PIG	LP2
60S ribosomal protein L12	Q6QAS5_PIG	LP2	Glutathione transferase ζ 1	F1S2N0_PIG	LP1
60S ribosomal protein L22	RL22_PIG	LP2	Heterogeneous nuclear ribonucleoprotein K	I3LQS0_PIG	LP2
60S acidic ribosomal protein P1	F1SIT7_PIG	LP2	Proteasome subunit α type-2	I3LAB6_PIG	LP2
ABI gene family member 3 isoform 2	I3LB01_PIG	LP2	protein-arginine deiminase type-2	I3LNE4_PIG	LP2
AP-1 complex subunit sigma-1A	Q06AS6_PIG	LP2	Ribosomal phosphoprotein large PO subunit	RLA0_PIG	LP2
cAMP-dependent protein kinase type II-α regulatory subunit	KAP2_PIG	LP1	Protein kinase C ε type	F1S5K7_PIG	LP2
Creatine kinase	I3LPB5_PIG	LP1, LP2	S100 Ca^2+^ binding protein A16	F2Z5M4_PIG	LP2
Cytoplasmic dynein 1 heavy chain 1	F1S9Y5_PIG	LS2, LP2			
***Nucleus***					
Barrier-to-autointegration factor-like isoform 1	F1RU33_PIG	LP1	Nucleosome assembly protein 1	F1SGD7_PIG	LP2
Dynein light chain Tctex-type 3	I3LUI9_PIG	LP2	WD repeat-containing protein 61	F1RKU4_PIG	LP2
Homeobox prox 1	F1SFF4_PIG	LP2			
***Vesicle***					
Membrane-associated progesterone receptor component 1	PGRC1_PIG	LP2	Syntaxin-1A	F1RJM9_PIG	LP2
Synaptic vesicle glycoprotein 2B	F1SCI2_PIG	LP2	Vacuolar protein sorting-associated protein 45	F1SDG5_PIG	LP2
Synaptotagmin-1	F1RYK1_PIG	LP2	V-type proton ATPase subunit D	F1SA40_PIG	LP2
			Vesicle-associated membrane protein-associated protein B	VAPB_PIG	LP2
***Cytoskeleton***					
Actin related protein 2/3 complex, subunit 4	F1SQE6_PIG	LP2	Dynamin-1	F1RRW8_PIG	LP2
Actin-related protein 2/3 complex subunit 5	B5APV0_PIG	LP2	Erythrocyte membrane protein band 4.1	F1SM86_PIG	LP2
Actin-related protein 3	F2Z5D2_PIG	LP2	Microtubule-associated protein 1B	F1SK12_PIG	LP1
Adenylyl cyclase-associated protein 1	I3LVT1_PIG	LP2	Microtubule-associated protein 2 isoform 2	F1SSS6_PIG	LS1, LP1
Cytoskeleton-associated protein 4	F1SPP8_PIG	LP2	Septin 2	F1SIP0_PIG	LS1
***Secreted***					
Granulin	F1RQZ0_PIG	LP1, LS2			

Co-immunoprecipitations of proteins from detergent extracts of the synaptosomal fractions from porcine brain using recombinant αSN monomer, αSN oligomer, or buffer as bait, were conducted, and the interacting proteins were identified by LC-LTQ orbitrap MS/MS. The identified proteins are specified by name, Uniprot/swiss-Prot entry, primary accession number, and the synaptosomal fraction in which they were identified. Quantitative analyses were based on Peak Area Intensity on peptides occurring in at least two of three individual immunoprecipitations. The presented as αSN binding proteins without any apparent conformational preferences are those where the ratio between peak areas for monomer and oligomers were < 2.

### Validation of protein targets by immunoblotting

Antibodies targeting the proteins selected for validation were tested by immunoblotting on all four subcellular fractions and the fraction with the highest signal was used as source for the validating co-IP (S3 Fig.). Proteins were resolved on 10–16% gradient SDS-PAGE under reducing conditions followed by immunoblotting, as described previously [45]. Quantification of band intensity on western blot was conducted in Image J (Research Services Branch, http://rsb.info.nih.gov/ij/index.hmtl). For the anti-αSN immunoblot in Fig. 1C-D, the filter was fixed in 0.4% PFA for 30 min at room temperature to reduce dissociation from the PVDF membrane, and boiled in PBS for 5 min prior to blocking followed by conventional immunoblotting procedure [46].

### Antibodies

Poly-clonal rabbit anti-αSN, ASY-1 and the αSN aggregation-specific Fila-1 were produced in house [22, 45, 47, 48]. Rabbit polyclonal anti-GFAP (Z0334) was purchased from Dako. Rabbit polyclonal anti-Glutamate decarboxylase 2 (ab49832), rabbit polyclonal anti-Synapsin I (ab64581), rabbit polyclonal anti-synaptophysin (ab8049), rabbit polyclonal anti-α-VDAC1 (ab15895), and anti-LAMP2a (ab18528) were purchased from Abcam. Mouse monoclonal anti-Abl Interactor 1 (MABS273) was purchased from Milipore. Mouse monoclonal anti-myelin proteolipid protein (ST-MCA839G) was purchased from AbD Serotec/BIO RAD. Mouse monoclonal anti-BiP/GRP78 (610979) and mouse monoclonal anti-αSN (BD, 610787) were purchased from BD Transduction Laboratories™. The rabbit polyclonal anti-VAMP-2 antibody was produced as previously described [49].

## Results

αSN oligomers can be formed *in vitro*in various ways e.g. oxidatively modified by dopamine [50]. We isolate spontaneously assembled, non-covalently modified αSN oligomers by gel filtration [41], which display the FILA-1 epitope that is shared with oligomers present in human dementia with Lewy body brain tissue and αSN transgenic mouse [21–23]. Such oligomers are structured but readily depolymerized upon denaturation [51]. Fig. 1A demonstrates the oligomers elute between 17 and 22 min and this pooled fraction is designated O. The monomers elute between 32 min and 37 min and the fraction collected at 35 min, designated M, was used for the further investigations. The presence of aggregate specific epitopes on oligomers, but not monomers, are demonstrated by their binding of the antibody Fila-1 (Fig. 1B), which bind an epitope that is shared with mature fibrils and occur in pathological αSN aggregates in brain [21–23].

αSN is predominantly a presynaptic protein so we investigated fractions of brain synaptosomes rather than crude brain homogenates in search for potential binding partners for exogenous monomeric and oligomeric αSN. The non-denaturing detergent Triton X-100 (0.5%) was used to extract the four subfractions, synaptosomal membranes (LP1), soluble synaptosomal lysate (LS1), and its subfractions synaptic vesicles (LP2) and synaptosomal cytosol (LS2) (S1 Fig.). The sub-fractionation was conducted to reduce the complexity of the samples in order to favor the downstream MS based identification of proteins co-purifying with αSN or buffer control. The composition of the sub-fractions was analyzed by immunoblotting for proteins known to be present in specific compartment. Synaptophysin, a marker for synaptic vesicles, was enriched in LP2, but could also be detected in LS1 and LP1 (Fig. 1C). The content of endoplasmic reticulum, was analyzed by the presence of 78 kDa glucose-regulated protein (GRP78), and GRP78 was also enriched in LP2, and detectable in LS1, LP1, and LS2. The mitochondrial content was visualized by voltage-dependent anionic channel (VDAC) and found exclusively in the LP1 fraction. Lamp2a, a marker for lysosomes, was enriched in LP2. Furthermore, all the synaptosomal fractions contained α-tubulin.

First, we analyzed whether the oligomers and monomers were stable during the procedure of co-IP by comparing their gel filtration behavior before and after incubation for 24 h at 4^o^C in the detergent containing synaptosomal cytosol (2µg/ml recombinant monomer or oligomer αSN in 1 mg/ml extract) as determined by anti-αSN immunoblotting of fractions corresponding to O, and M (Fig. 1D). A fraction (I) eluting at 25 min, which is expected to contain low amounts of exogenous αSN (Fig. 1A), was also analyzed in order to investigate a dissociation of the oligomer into smaller species. The endogenous αSN level in the LS2 fraction was low, compared to samples supplemented 2µg recombinant monomer or oligomer αSN, but can be detected in the lysate sample without recombinant αSN primarily eluting as monomer (Fig. 1D). The exogenous monomer eluted in the M fraction after incubation for 24 h with small amounts of high molecular weight material present in fraction I. The oligomers also retained their elution in the high molecular weight fraction O, but with some dissociation into complexes eluting in fraction I and with little detectable material in the fraction M, likely corresponding to endogenous porcine αSN. This shows that the purified oligomers and monomers largely retain their molecular sizes during the incubation in detergent extract of brain lysates for 24 h at 4°C.

For proteomic analysis the brain extracts were incubated with recombinant human αSN in its monomeric, and oligomeric state, or buffer-control followed by pull-down using the polyclonal αSN antibody ASY-1 coupled to sepharose beads. Co-IP proteins were identified by tryptic peptide mapping by LC-MS/MS. Three identical experiments were conducted using the four different synaptosomal preparations and each with monomeric, oligomeric αSN, or buffer control as bait. The 36 individual co-IPs were analyzed by LC-LTQ orbitrap tandem mass spectrometry, which returned 11087 identifying peptides corresponding to 2049 proteins by searching of a *Sus scrofa* database. The criterion to be fulfilled for proteins to be considered as potential binding partners was identification in at least two of the three biological samples. To compare if binding of a protein is preferential to αSN monomer or αSN oligomer, semi-quantitative data was obtained as the peak area. The peak area of individual peptides varied considerably between runs as exemplified by the peak area from ELEAVCQDVLSLLDNYLIK peptide of 14–3–3 protein gamma that ranged from 397,866 to 1,415,206 in buffer controls, from 509,836 to 724,293 in monomer samples and from 1,439,298 to 9,995,353 in oligomer samples. In order to normalize the individual runs, a logarithmic transformation of the MS peak area output was conducted before comparing the runs. This resulted for the ELEAVCQDVLSLLDNYLIK peptide in control values from 5.6 to 6.15, monomer values from 5.71 to 5.86 and oligomer values from 6.16 to 7.0 thus demonstrating a significant difference (p-value 0.03) between oligomer and monomer or buffer control when analyzed by student’s t-test. A graphical illustration of the sample preparation, analysis and semi-quantitative analysis and the criteria used can be found in S2 Fig.

A total of 178 proteins fulfilled the criteria of being αSN-binding proteins that are present significantly higher in αSN fractions than in buffer controls. 87% of the αSN-binding proteins occurred uniquely among one of the fractions. Tubulin beta-4 and synapsin 1 were present in three fractions and only reticulocalbin 2 was present in all four fractions.

The αSN binding proteins were filtered for their preferential occurrence in oligomer or monomer samples based on the calculated average fold increase between the samples or those being evenly distributed in these fractions. This resulted in lists of preferential monomer-binding proteins (MP; Table 1), preferential oligomer-binding proteins (OP, Table 2) and those not displaying preference between the αSN forms (NPB; Table 3). The tables also display the predicted subcellular localization of the proteins based on Uniprot KB database.

**Figure 2:**
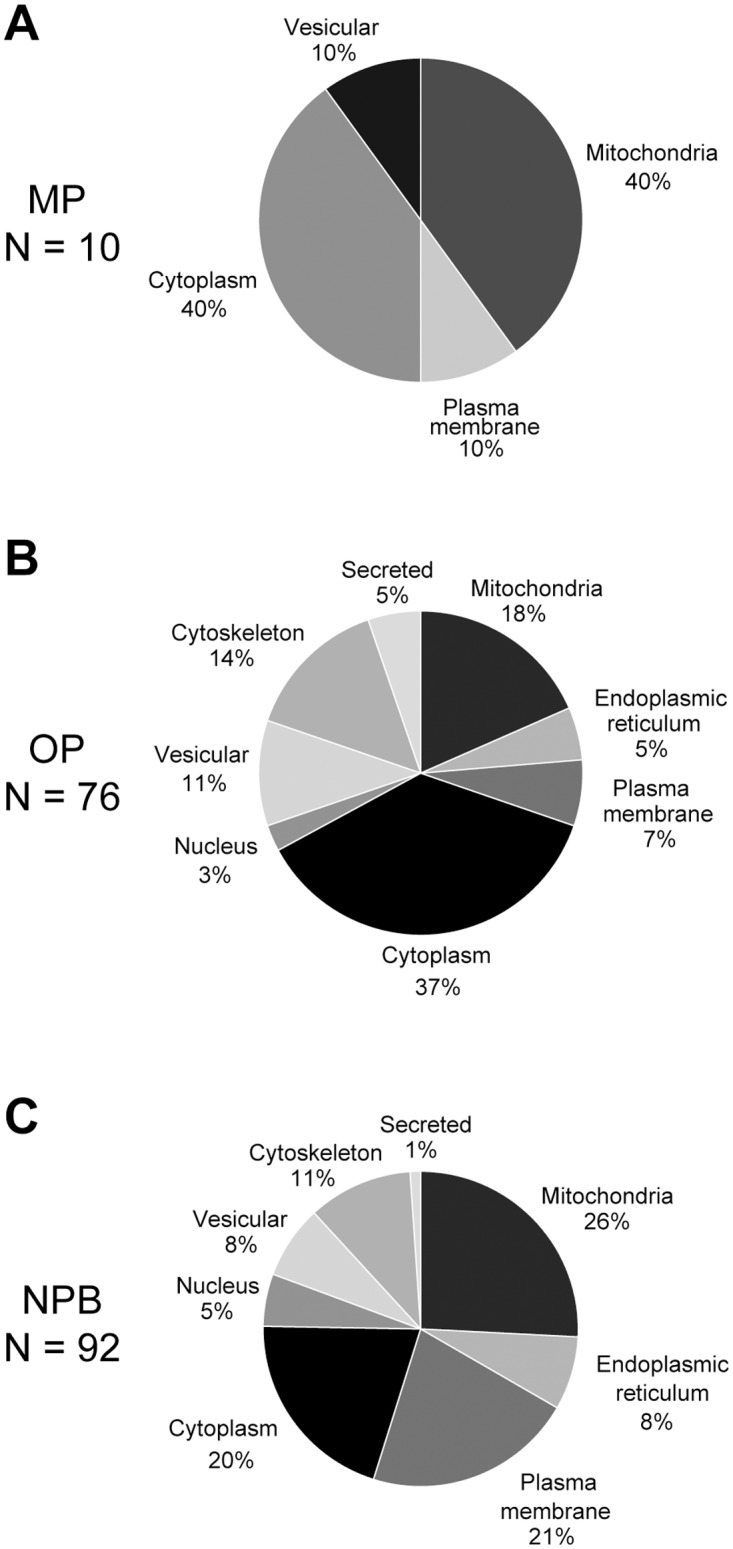
Subcellular localization of the αSN interacting proteins. A total of 178 proteins were identified as αSN interacting proteins and they were grouped in proteins preferentially binding monomer αSN (MP, N = 10, Table 1), oligomers (OP, N = 76, Table 2) and proteins not displaying any preferences (NPB, N = 92, Table 3). They were grouped based on their subcellular localization as described by their principal localization in the Uniprot database to demonstrate the aggregation state of αSN have potential for significantly changing its cellular targets.

Ten preferential monomer-binding proteins (Table 1) were identified and their predicted subcellular localizations are dominated by mitochondria and cytosol (Fig. 2A). It should be kept in mind that the detergent extraction removes all membranes and thus binding of e.g. intra-mitochondrial proteins must be interpreted with caution.

The preferential oligomer-binding proteins (Table 2) comprised a large group of 76 that displayed a complex subcellular distribution although it was dominated by cytosolic and cytoskeletal proteins that may be immediately accessible to cytosolic αSN albeit a large group of mitochondrial proteins were also present (Fig. 2B). It was reassuring to identify the protein p25α/tubulin polymerization promoting protein in this group because it previously has been identified as a preferential binder to aggregated αSN species [48].

The non-preferential group was the largest with 92 proteins (Table 3). Its subcellular distribution was complex and slightly more evenly distributed between the categories than the preferential oligomer-binding protein (Fig. 2C).

**Figure 3:**
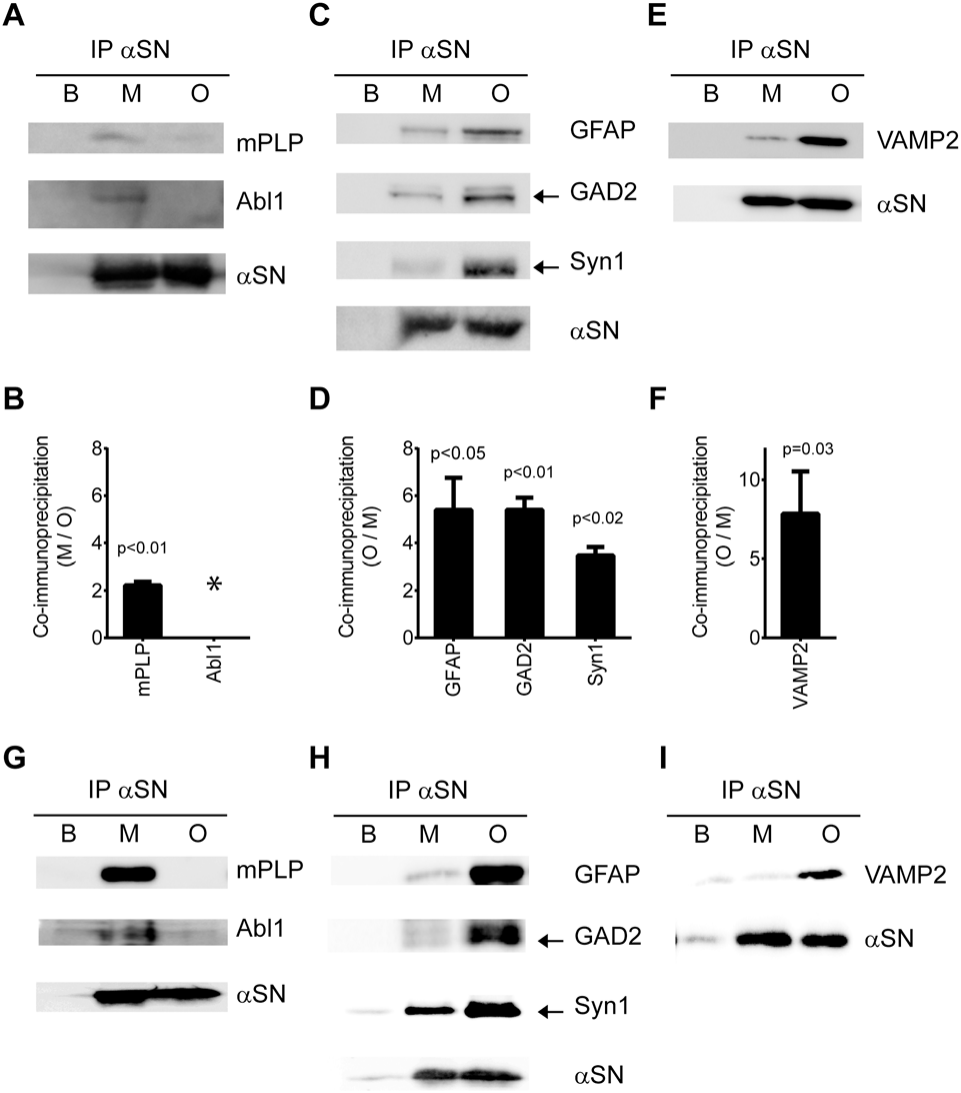
Validation of monomer and oligomer preference of αSN interacting proteins. Proteins pulled down by monomer αSN (M), oligomer αSN (O), and buffer control (B) from porcine (A-F) and human (G-I) brain extracts were analyzed by immunoblotting using antibodies against antigens selected among the monomer and oligomer binding proteins. Monomer binding antigens were myelin proteolipid protein (mPLP) and Abl interactor 1 (Abl1) and oligomer binding proteins were glial fibrillary acidic protein (GFAP), glutamate decarboxylase 2 (GAD2), and synapsin 1 (Syn1). VAMP-2 was tested because it has been reported to bind αSN, although it was not detected in our proteomic analysis. One representative of three experiments is presented for porcine αSN binding proteins (A, C, E), and the quantification of the three experiments is presented in panels B, D, F. The quantification of bands was performed after subtracting the non-specific signal in the buffer control from the specific bands in monomer and oligomer samples. Bars represent mean ratio between monomer and oligomer ± S.D. of the three replicates. The values for binding to monomer and oligomer were compared by Student’s t-test and the resulting p-values are listed above the bars. * Indicates that the band intensity from oligomer did not differ significantly from background making quantifications impracticable. In order to ensure that the interaction were not due to species differences between human and porcine proteins we conducted validations in human brain extracts. One representative of two experiments is presented for each validated protein. The validation for both porcine and human of mPLP, Abl1, Syn1 and VAMP-2 was conducted in the LP2 fraction enriched in synaptic vesicle and the validation to GFAP and GAD2 in the LS1 fraction of synaptosomal lysate.

The increased binding of proteins to oligomers and monomers are displayed by the fold-increase of binding to the corresponding αSN form (oligomer vs. monomer) based on the average of all their identified peptides (Tables 1 and 2). These values varied greatly from 2 to 802 and thus call for caution when interpreting the increase for proteins that are based on few peptides. To corroborate their qualitative nature, we performed additional Co-IP experiments followed by immunoblotting for selected proteins. Proteins for validation were selected based on availability of well-characterized antibodies raised against human epitopes with at least 95% identity to the porcine protein. As representatives for the preferential monomer-binding proteins, Myelin proteolipid protein (mPLP) and Abl interactor 1 (Abl1) were chosen (Fig. 3A, B) and for preferential oligomer-binding proteins, Glial fibrillary acidic protein (GFAP), Glutamate decarboxylase 2 (GAD2), and Synapsin 1 (Syn1) were chosen (Fig. 3C, D). The data confirm the αSN conformation preference identified by the MS-based technique. The quantitative analyses of three individual pull-down experiments were conducted and resulted in approximately 4–6 times increased binding to oligomers vs. monomers for the oligomer preferential proteins. The results for the monomer binding proteins also clearly demonstrated the preference for monomers although the absolute value for the monomer preference of Abl 1 is unclear because the oligomer binding was as low as the buffer control thus making estimation of ratios pointless.

αSN has been demonstrated as a chaperone for Vesicle-associated membrane protein 2 (VAMP-2), also named synaptobrevin-2, in nerve terminals [7] so it was a surprise that we did not detect VAMP-2 in our proteomic screen. However, inspection of the potential tryptic peptides generated from VAMP-2 demonstrates that only 2 recognizable peptides can be formed. Hence, such peptides may have been missed in the MS-based peptide search. Fig. 3E demonstrates a co-IP analysis of the VAMP-2 binding to monomer and oligomer αSN. This clearly demonstrates that oligomerization αSN enhances the interaction. In order to ensure that the interactions were not artifacts due to species differences between human αSN and porcine brain synaptosomal preparations, we performed validative Co-IPs using human brain synaptosomal preparations, and were able to confirm the finding from porcine brain synaptosomal extracts (Fig. 3G-I).

## Discussion

**Figure 4:**
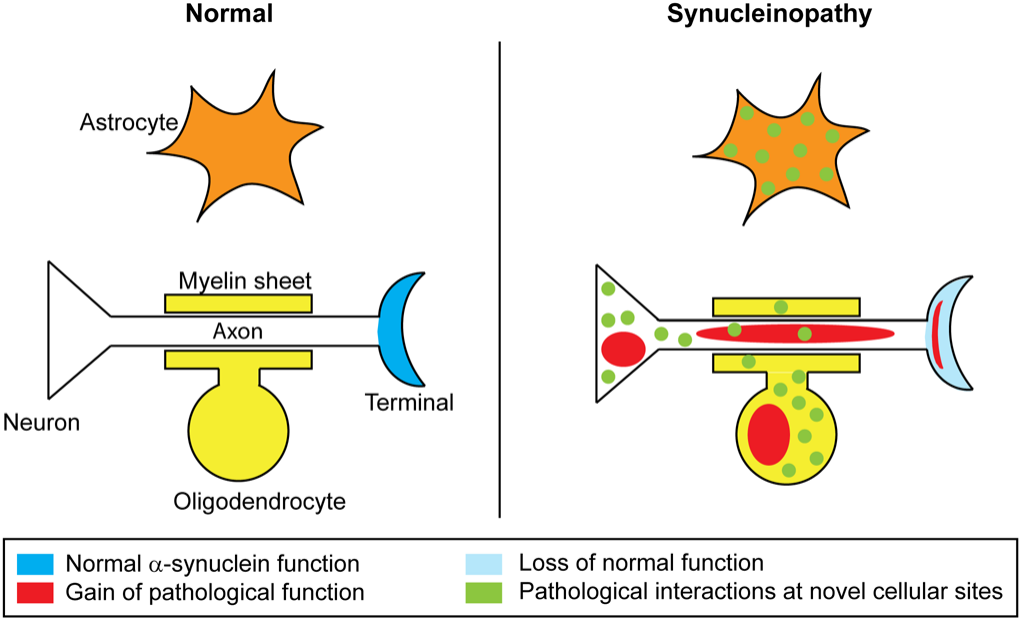
Possible molecular pathways initiated by αSN in disease. Under normal conditions αSN is predominantly located in nerve terminals (blue). During disease αSN undergo aggregation and this lead to novel conformation-dependent interactions (red), which represents a gain of function. In addition, αSN species (monomeric and oligomeric) are concentrated at abnormal sites, like axons and the cell body, or in astrocytes and oligodendrocytes, which give rise to novel interactions because new partners are introduced (green). Finally, an abnormal sorting and aggregation leads to a loss of, or reduced normal αSN concentration in nerve terminals where critical monomer specific interactions will be compromised thus representing a loss of function.

The functionality of αSN in neurons is related to its state of aggregation. Native monomeric species are hypothesized to conduct physiological roles in nerve terminals, where αSN is present at high concentrations. In brain tissue affected by PD and DLB, αSN accumulates as soluble oligomeric or protofibrillar species in terminals, axons and the somatodendritic compartment and filamentous in Lewy inclusions and Lewy neurites. Moreover, αSN is also present in astrocytes in PD [52] and oligodendrocytes in MSA [53]. Hence, at least three factors have to be considered for possible molecular pathogenic pathways initiated by αSN in disease (Fig. 4). First, a gain of function by novel structures on aggregated oligomeric species. Second, novel functions of αSN species, being monomeric and oligomeric, concentrated at abnormal sites like axons and the cell body or in astrocytes and oligodendrocytes, where they will encounter new interaction partners. Third, potential loss of function by the native monomer population if reduced due to aggregation or abnormal sorting.

We conducted a proteomic screen in sub-fractions from porcine synaptosomes to identify potential interacting networks for αSN that could be affected in α-synucleinopathies. The semi-quantitative proteomic screen was conducted using exogenous human αSN as bait in its monomeric and pathological oligomeric state. Many types of *in vitro* formed αSN oligomers have been reported, some forming spontaneously [41], some being stabilized by organic solvents [54], and chemical modifications like dopamine [50] and some being off- versus on-pathway in the aggregation process [55]. We choose to use the protocol for spontaneously formed oligomers, which we have characterized extensively by surface hydrogen/deuterium exchange combined with mass spectrometry and FILA-1 binding [36, 41, 51]. These oligomers share surface epitopes for the conformational specific antibody FILA-1 with αSN oligomers present in pathological human and mouse brain tissue [21–23]. It was imperative for the experiment that the monomer and oligomer αSN retained their gross molecular structure during the 24h period of the incubations in order to perform a pull-down analysis of ligands for the two conformations. This is especially critical because the non-modified oligomers we use display some dissociation into monomers [51]. Comfortingly, we could demonstrate that the majority of the exogenously added monomer and oligomer retained their sizes as determined by gel filtration of the detergent brain extracts after the 24 h incubation. The elution of monomeric input αSN as monomers after the incubation with brain extracts indicates most remains unbound or so loosely associated to targets that the complex dissociates during the gel filtration. The large and heterogeneous size of oligomers based on their gel filtration profile does not allow judgment of whether they have bound ligands or not by the subsequent gel filtration analysis.

Using the above pull-down protocol 178 proteins were enriched in the αSN containing samples compared to the buffer control according to our specified criteria. These proteins were selected from a total of 11087 MS spectra corresponding to 2049 proteins identified by searching a *Sus scrofa* database containing all canonical sequences plus all manually reviewed isoform sequences retrieved from UniProt (July, 2012). This number of spectra is comparable to the number of spectra identified in a previous proteomic screen in mouse brain synaptosomes for ligands of the C-terminal 40 residues of αSN [35]. We identified only 41 significantly enriched proteins in the soluble synaptosomal lysate compared to 121 in its two resulting subfractions, synaptic vesicles and synaptosomal lysate. This demonstrates the power of reducing the complexity in targeted proteomic searches but also highlights a limitation in deciphering complex interacting networks spanning cytosolic constituents and organelles. Of the 2049 identified proteins, 178 proteins had significant increased binding to αSN compared to background. The 178 proteins could be grouped in three categories using > 2 fold-increase as indicative of preferential binding. Preferential monomer binding proteins (MPB) are at risk of loosing their function when aggregation occurs. Preferential oligomer binding protein (OPB) may initiate novel conformation-dependent interactions and No-preference binding (NPB) are proteins that can form novel interactions if the cellular/subcellular context of αSN is changed. To validate the preferential binding to monomer, oligomer and buffer control by immunoblotting, we selected mPLP and Abl1 among the 10 MPB, and GFAP, GAD2 and Syn1 among the 76 OPB and obtained for all selected a positive validation of their predicted target preference. Three amino acids differs between human and porcine αSN, so in order to ensure that the identified interactions were not artifacts due to these differences, the interactions were all positively validated using human synaptosomal preparations. This supports the concept of groups of cellular proteins preferentially binding either monomeric or oligomeric αSN and thus representing interaction networks at risk of being offset in α-synucleinopathies. The larger number of identified interaction partners of OBP compare to MBP could be due to the unfolded nature of monomer αSN compared to oligomer that display distinct structural feature as demonstrated by antigen presentation [21] and deuterium exchange mass spectrometric analysis [51].

αSN is considered a predominantly cytosolic protein and cytosolic proteins also represent the largest group with 51 of 178 αSN binding proteins. However, it is evident that αSN species holds potential for targeting proteins in organelles like mitochondria, endoplasmic reticulum, vesicles, plasma membrane and cytoskeleton. As cautionary note, one must be aware that the use of detergents dissolve natural membrane barriers and may give rise to false positive interactions. With that mentioned, mitochondria have previously been reported to interact with and take up αSN [56, 57] and cellular αSN expression increases mitochondrial calcium uptake [58] and reduces mitochondrial membrane potential [59]. Our data support that αSN can modulate mitochondrial functionality as we confirm binding to the previously found mitochondrial chaperone mortalin [34] and identify novel targets e.g. in the outer mitochondrial membrane like voltage-dependent anion-selective channel protein 1 and translocase of outer mitochondrial membrane but also components involved in ATP production like ATP synthase subunits alpha and beta. Interestingly, ATP synthase subunit alpha is increased in αSN expressing SH-SY5Y cells [60].

Autophagy has attracted significant interest in PD research because of its ability to degrade intracellular proteins in general and misfolded proteins in particular [61] and because mutations in lysosomal glycocerebrosidase, causing Gaucher’s disease, increase the risk of developing PD [62]. Moreover, dysfunctional autophagic flux results in increased cellular excretion of αSN species, which have been sampled from the cytosol by quality control autophagy [63]. Such species may hold increased potential for spreading misfolded αSN to other cells compared to the normal constitutively excreted αSN. Tightly regulated vesicle sorting and acidification of vesicular content is critical for the autophagic flux and proper enzymatic degradation of its cargo. We find αSN hold potential for disturbing these processes because the all three αSN binding fractions contain subunits associated with acidifying proton pumps (V-type proton ATPase subunit D, D1 and F), along with vacuolar sorting proteins (vesicle-associated membrane protein-associated B; vacuolar protein sorting-associated protein 53).

αSN aggregates have been demonstrated in synaptic terminals of brain tissue affected by Lewy body dementia [25]. In the terminals oligomers may inhibit normal synaptic vesicle function because αSN oligomers display increased binding of synaptic vesicle proteins amphiphysin, synapsin 1, synaptic vesicle glycoprotein 2a and VAMP-2 (also known as synaptobrevin 2). αSN has been demonstrated to function as a critical chaperone for VAMP-2 function [7] and this function is hypothesized to rely on a tethering mediated by vesicle binding to the N-terminus and VAMP-2 binding to the C-terminus of αSN [64]. Oligomerization of αSN will enhance VAMP-2 binding and likely inhibit the ordered tethering mediated by native αSN. We did not identify VAMP-2 by our proteomic investigation, but could demonstrate its preferential binding to oligomers by co-IP. This highlights the limitations of proteomic studies where negative results have to be interpreted with care.

Calcium dysregulation is potentially involved in sporadic PD because treatment with calcium antagonists in epidemiological studies has demonstrated a protective effect [65]. We find that the type 4 plasma membrane Ca^2+^ ATPase displays increased binding of oligomeric αSN, whereas binding to type 2 plasma membrane Ca^2+^ ATPase displays no conformational preference. Additionally, we have in another pull-down study demonstrated increased binding of αSN oligomers to ER-associated Ca^2+^ ATPase and this interaction functionally activates the pump (Betzer, Jensen, Manuscript in preparation). Hence, aggregation and redistribution of αSN from its normal presynaptic localization to larger neuronal compartments hold the potential of dysregulating these important Ca^2+^ pumps, which are critical for restoring normal cytosolic calcium levels after locally increased Ca^2+^ transients involved in signaling. Likewise, dysregulation of excitatory amino acid transporter 1 by αSN oligomeric may affect the removal of glutamate from the synaptic cleft [66] and thus cause hyperactive glutamate signaling and potentially excitotoxicity.

Dysregulation of protein translation has recently been associated to PD by the demonstration of mutations in the translation initiation factor EIF4G1 in familial PD [67] and the identification of specific R1205H variant as a PD risk factor [68]. We did not identify EIF4G1 or its directly interacting partners, but demonstrate several proteins associated to the subsequent steps in protein translation, elongation factors 1 alpha and beta, 40S ribosomal proteins S3, S3a, S5-like protein isoform 2, 60S proteins L6, L12, L22, L32 and acidic ribosomal protein 3-like isoform 2. Among these interactions are both aggregate and non-aggregated dependent interactions. Association of dysregulated protein synthesis in PD was thus initially highlighted by the EIF4G1 mutations [67], but our findings suggest that PD causing defects in protein synthesis can be triggered by dislocalized or misfolded αSN at other levels in this biosynthetic pathway.

Potential targets in signaling cascades were also identified. The cAMP and Protein kinase A (PKA) signaling system may be affected at more levels by interactions with catalytic subunit beta, regulatory subunit type 2, as well as scaffolding A-kinase anchor protein 5. Such interactions may be involved in the αSN dependent attenuation of cAMP dependent TH gene expression in cells [69]. Additionally, we identified Protein kinase C epsilon, calcium/calmodulin-dependent protein kinase type 2, alpha subunit and creatine kinase thus linking αSN sensitive targets to phospholipase C and its substrate diacylglycerol, calcium regulation and bioenergetics.

Unfolded protein stress in an αSN transgenic mouse, was associated to accumulation of misfolded αSN species in the endoplasmic reticulum of brain tissue and such accumulation was also demonstrated in humans brains affected by α-synucleinopathies [22]. Our demonstration of protein disulfide-isomerase A6 binding to αSN could provide a mechanistic link between accumulated αSN aggregates and interference with protein disulfide bridge formation, folding and ER stress.

The strong preference for sirtuin 2 binding to oligomeric αSN may indicate direct protein-protein interactions as involved in the role of protein deacetylase sirtuin 2 in nigrostriatal damage in toxic and αSN transgenic PD models [70, 71].

## Conclusion

We identify a range of αSN interacting brain proteins among which several displays monomer and aggregate specific preferences. The identified ligands validate proteins previously reported to interact with αSN (including actin [33], tubulins [72, 73], p25α [48], and mortalin[34]), but also highlights novel proteins from pathways already hypothesized to be involved in the molecular pathogenesis of α-synucleinopathies like calcium regulation, mitochondrial homeostasis, protein translation, kinase signaling and vesicular and lysosomal function. Hence, the data may both corroborate current hypotheses by expanding their involved targets and contribute to the generation of novel hypotheses.

## Supporting Information

S1 Fig Fractionation process for generating the synaptosomal fractions for the interaction analysis. Porcine and human brain tissue was homogenized and subjected to differential centrifugation as described in Materials and Methods. Enclosed in the dashed box is the four synaptosomal preparations; synaptosomal membranes (LP1), synaptic lysate (LS1), synaptic vesicles (LP2), and synaptic cytosol (LS2), which were used in the co-immunoprecipitations and subsequent identifications of αSN binding proteins upon solubilized in 0.5% Triton X-100.
(TIF)

S2 Fig Schematic illustration of the workflow and decision making used in the semi-quantitative mass spectrometric analysis. Brain tissue is prepared and fractioned resulting in four synaptosomal fractions (LP1, LS1, LP2, and LS2) before being mixed with either αSN monomer, oligomer, or buffer control yielding 12 samples. By conducting the investigation on three biological replicates this gives a total of 36 samples. The 36 samples were processed by co-IP, tryptic digestion, fractionation and analysis by LC-LTQ orbitrap tandem mass spectrometry. Peptides identified in at least two of the 36 samples were analyzed by integrating the peak area intensity (PAI) using Skyline to obtain semi-quantitative information. The semi-quantitative data was logarithmic transformed to reduce variation between biological replicates. All the quantitative data were manually inspected for peptides with higher buffer values compared to monomer or oligomer values and these peptides were excluded. To determine conformation preferences among identified ligand interactions a ratio was calculated between monomer signal and oligomer signal for each of the three biological replicates. The significance was tested by Students t-test and significant average fold increase of 2 or more designates conformation preferential binding.
(TIF)

S3 Fig Presence of αSN binding proteins chosen for validation in synaptosomal fractions. 30µg of each of the fractions synaptosomal membranes LP1, synaptosomal lysate (LS1), synaptic vesicles (LP2) and synaptosomal cytosol (LS2) were immunoblottet, and analyzed for the presence of myelin Proteolipid protein (mPLP), Abl interactor 1 (Abl1), Glial fibrillary acidic protein (GFAP), Glutamic acid decarboxylase 2 (GAD2), Synapsin 1 (Syn1), and Vesicle associated membrane protein 2 (VAMP2).
(TIF)

S1 Table Semi-quantitative data from mass spectrometric analysis. semi-quantitative analysis of protein binding to alpha-synuclein, subsectioned into three conditions, monomer binding proteins, oligomer binding proteins, and no difference in binding monomer and oligomer including number of identified peptides, number of quantified peptides, and percent sequence coverage.
(XLSX)
