# Supplementary figures and images for: Identification of Synaptosomal Proteins Binding to Monomeric and Oligomeric α-Synuclein
Source: PLoS One. 2015 Feb 6;10(2):e0116473. doi: 10.1371/journal.pone.0116473 (PMC4319895; doi:10.1371/journal.pone.0116473)

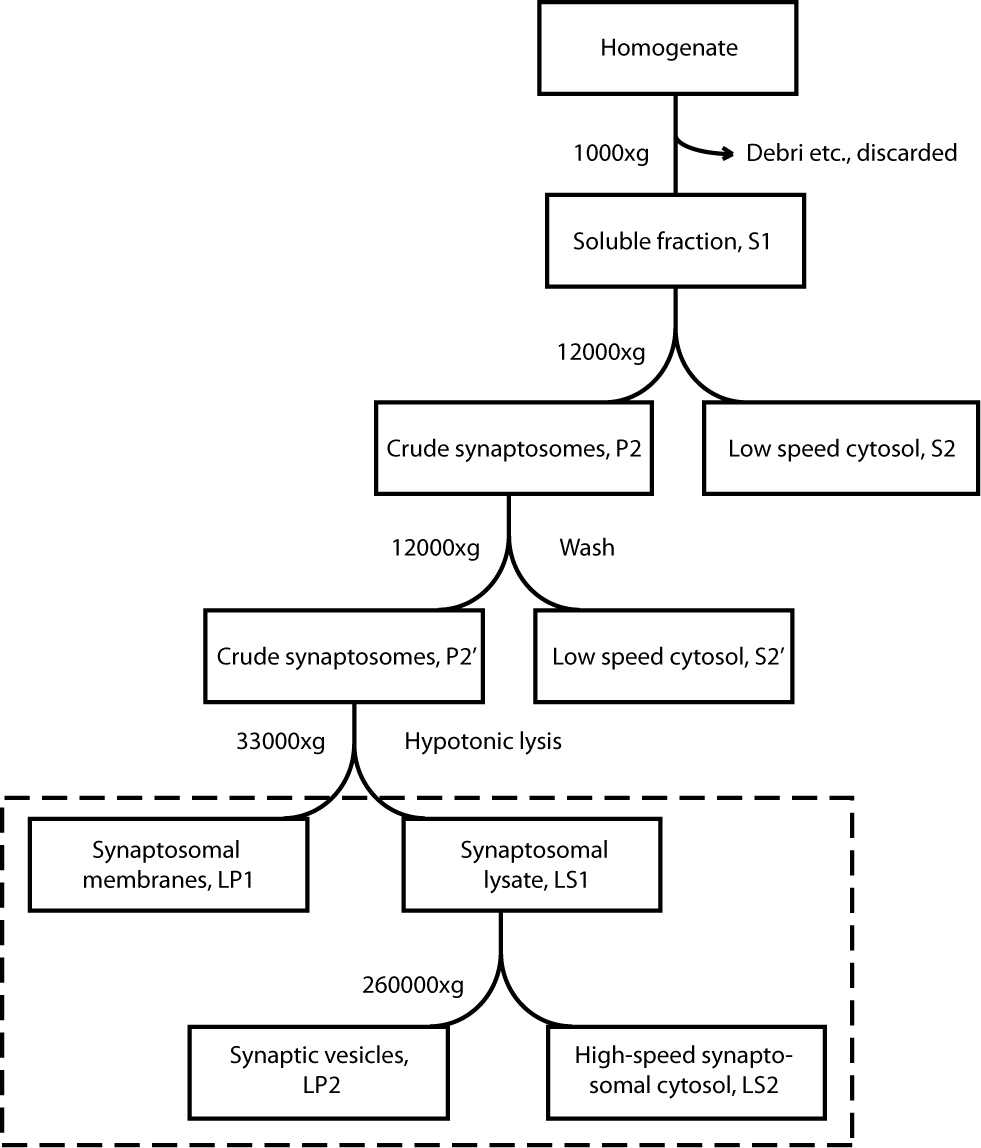

Supplement: S1 Fig — Porcine and human brain tissue was homogenized and subjected to differential centrifugation as described in Materials and Methods. Enclosed in the dashed box is the four synaptosomal preparations; synaptosomal membranes (LP1), synaptic lysate (LS1), synaptic vesicles (LP2), and synaptic cytosol (LS2), which were used in the co-immunoprecipitations and subsequent identifications of αSN binding proteins upon solubilized in 0.5% Triton X-100. (TIF) [file pone.0116473.s001.tif]

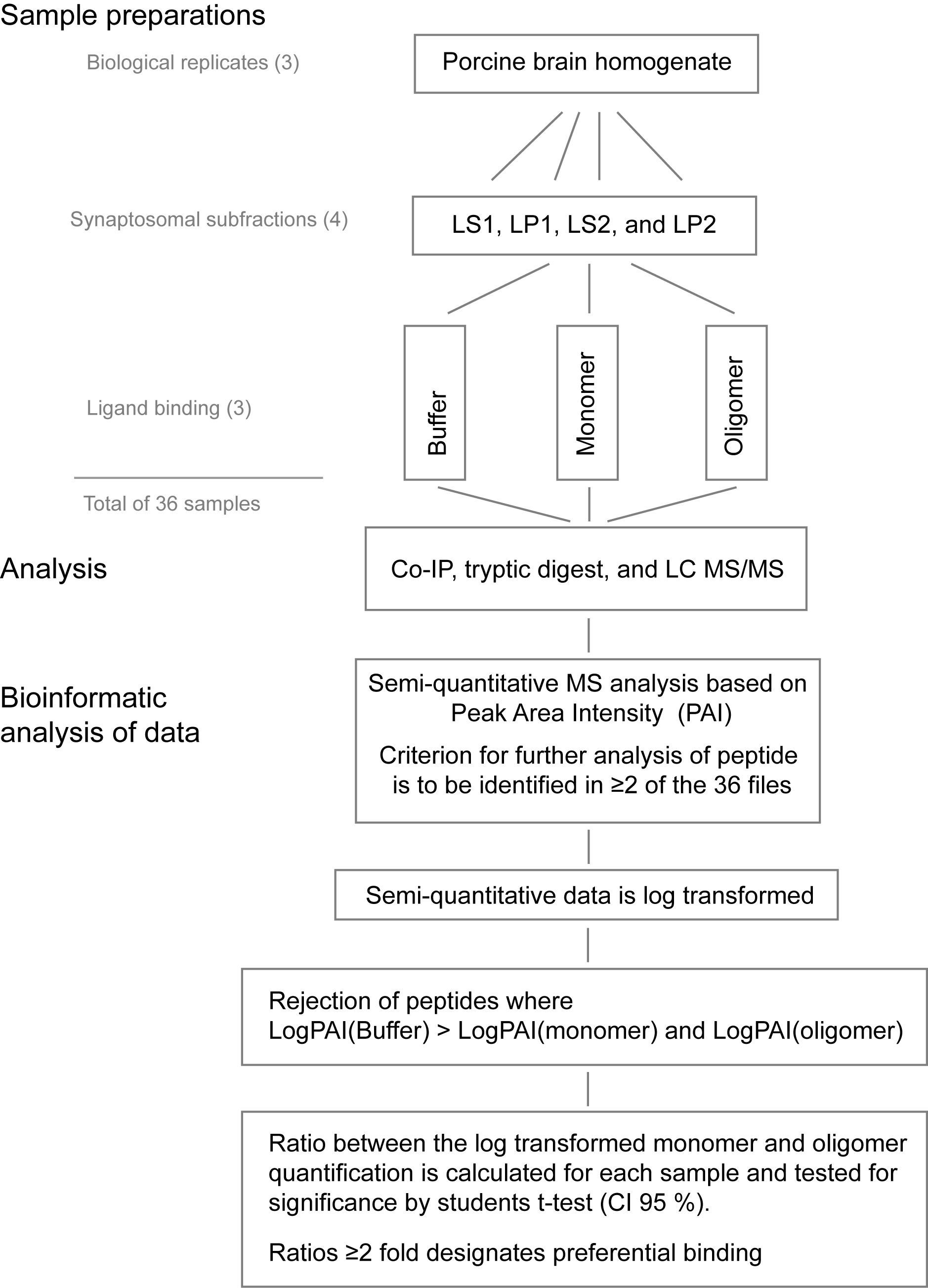

Supplement: S2 Fig — Brain tissue is prepared and fractioned resulting in four synaptosomal fractions (LP1, LS1, LP2, and LS2) before being mixed with either αSN monomer, oligomer, or buffer control yielding 12 samples. By conducting the investigation on three biological replicates this gives a total of 36 samples. The 36 samples were processed by co-IP, tryptic digestion, fractionation and analysis by LC-LTQ orbitrap tandem mass spectrometry. Peptides identified in at least two of the 36 samples were analyzed by integrating the peak area intensity (PAI) using Skyline to obtain semi-quantitative information. The semi-quantitative data was logarithmic transformed to reduce variation between biological replicates. All the quantitative data were manually inspected for peptides with higher buffer values compared to monomer or oligomer values and these peptides were excluded. To determine conformation preferences among identified ligand interactions a ratio was calculated between monomer signal and oligomer signal for each of the three biological replicates. The significance was tested by Students t-test and significant average fold increase of 2 or more designates conformation preferential binding. (TIF) [file pone.0116473.s002.tif]

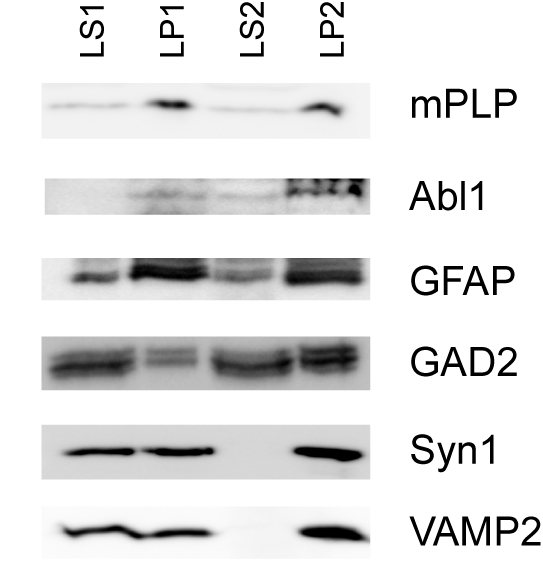

Supplement: S3 Fig — 30µg of each of the fractions synaptosomal membranes LP1, synaptosomal lysate (LS1), synaptic vesicles (LP2) and synaptosomal cytosol (LS2) were immunoblottet, and analyzed for the presence of myelin Proteolipid protein (mPLP), Abl interactor 1 (Abl1), Glial fibrillary acidic protein (GFAP), Glutamic acid decarboxylase 2 (GAD2), Synapsin 1 (Syn1), and Vesicle associated membrane protein 2 (VAMP2). (TIF) [file pone.0116473.s003.tif]
